# pH-Dependant Antifungal Activity of Valproic Acid against the Human Fungal Pathogen *Candida albicans*

**DOI:** 10.3389/fmicb.2017.01956

**Published:** 2017-10-09

**Authors:** Julien Chaillot, Faiza Tebbji, Carlos García, Hugo Wurtele, René Pelletier, Adnane Sellam

**Affiliations:** ^1^Infectious Diseases Research Centre-CRI, Research Center of the CHU de Québec, Université Laval, Quebec, QC, Canada; ^2^Maisonneuve-Rosemont Hospital Research Center, Montreal, QC, Canada; ^3^Department of Medicine, Université de Montréal, Montreal, QC, Canada; ^4^Medical Microbiology and Infectious Diseases, Research Center of the CHU de Québec, Quebec, QC, Canada; ^5^Department of Microbiology, Infectious Disease and Immunology, Faculty of Medicine, University Laval, Quebec, QC, Canada

**Keywords:** *Candida albicans*, valproic acid, antifungal, vacuole, vulvovaginal candidiasis

## Abstract

Current antifungal drugs suffer from limitations including toxicity, the emergence of resistance and decreased efficacy at low pH that are typical of human vaginal surfaces. Here, we have shown that the antipsychotic drug valproic acid (VPA) exhibited a strong antifungal activity against both sensitive and resistant *Candida albicans* in pH condition similar to that encountered in vagina. VPA exerted a strong anti-biofilm activity and attenuated damage of vaginal epithelial cells caused by *C. albicans*. We also showed that VPA synergizes with the allylamine antifungal, Terbinafine. We undertook a chemogenetic screen to delineate biological processes that underlies VPA-sensitivity in *C. albicans* and found that vacuole-related genes were required to tolerate VPA. Confocal fluorescence live-cell imaging revealed that VPA alters vacuole integrity and support a model where alteration of vacuoles contributes to the antifungal activity. Taken together, this study suggests that VPA could be used as an effective antifungal against vulvovaginal candidiasis.

## Introduction

*Candida albicans* is the major human fungal pathogens and also a component of the normal human flora, colonizing primarily mucosal surfaces, gastrointestinal and genitourinary tracts, and skin (Berman and Sudbery, 2002). Although many infections involve unpleasant but non-life-threatening colonization of various surface of mucosal membranes, immunosuppressed patients can fall prey to serious mucosal infections, such as oropharyngeal candidiasis in HIV patients and newborns, and lethal systemic infections (Odds, 1987). *C. albicans* followed by *C. glabrata* are natural components of the vaginal fungal microbiota and, opportunistically, the leading causative agents of vulvovaginal candidiasis (VVC). VVC affects 70–75% of childbearing women at least once, and 40–50% of them will experience recurrence (Sobel, 2007).

Topical azoles-based antifungal formulations (e.g., fluconazole, clotrimazole, miconazole, or butoconazole) such as vaginal suppositories, tablets, and cream are widely used to treat VVC. However, their efficiency is questioned especially for *C. glabrata* who is intrinsically resistant to azoles. Furthermore, VVC are often caused by *C. albicans* azole-resistant strains (Sobel, 2007; Marchaim et al., 2012). Importantly, antifungals used for VVC treatments had to fulfill the constraint of remaining effective at acidic pH (4–4.5), which is the normal pH of human vaginal surfaces. Recent studies had proven that the acidic pH increases the minimal inhibitory concentrations (MICs) of several antifungals including azoles, amphothericin B, ciclopirox olamine, flucytosine, and caspofungin for *C. albicans* (Danby et al., 2012). Pai and Jones reported a similar finding in *C. glabrata* where MICs of triazoles were increased in pH 6 as compared to pH 7.4 (Pai and Jones, 2004). Taken together, these data demonstrate that in addition to the complications related to the acquired or the intrinsic-resistance to conventional antifungals, reduction of antifungal potency at acidic pH can further complicate the treatments of VVC. Due to the fact that the antifungal discovery pipelines of pharmaceutical companies are almost dry, there is an urgent need to identify novel low pH-effective antifungal molecules for VVC therapeutic intervention.

Valproic acid (VPA), is a branched short-chain fatty acid well-known as a class I/II histone deacetylase inhibitor (HDACi) (Gottlicher et al., 2001; Phiel et al., 2001). VPA is widely prescribed as antipsychotic to treat epilepsy, bipolar disorder, and uncontrolled seizures (Privitera et al., 2006). The antifungal properties of VPA has been previously reported against different opportunistic fungi causing infections of the central nervous system (Galgoczy et al., 2012; Homa et al., 2015). Despite the growing interest on VPA as antifungal, its precise mechanism of action remains not clear. Recent investigations in the budding yeast *Saccharomyces cerevisiae* have shown that VPA induces apoptosis and inhibits both cell-cycle at the G1-S transition and the activation of the cell wall integrity pathway, Stl2 MAP kinase (Mitsui et al., 2005; Desfosses-Baron et al., 2016). VPA was also shown to cause inositol depletion which in turn led to vacuolar ATPase perturbation (Ju and Greenberg, 2003; Deranieh et al., 2015). In *Schizosaccharomyce pombe*, VPA acts as an HDACi and disturbs different cellular processes including calcium homeostasis, cell wall integrity, and membrane trafficking (Miyatake et al., 2007; Zhang et al., 2013).

We have recently shown that low pH strongly potentiates VPA antimicrobial activity against the model yeast *S. cerevisiae* (Desfosses-Baron et al., 2016). Here, we investigated the *in vitro* susceptibility of both planktonic and sessile cells of different sensitive and resistant clinical isolates of the opportunistic yeast *C. albicans* to VPA using conditions mimicking the vaginal environment. The effect of VPA on the ability of *C. albicans* to cause damage to vaginal epithelial cells were investigated. Drug synergy between VPA and 11 standard antifungal agents were also explored. In attempt to gain insight into the mechanism of action associated with the antifungal activity of VPA a genetic screen was undertaken to uncover mutations conferring hypersensitivity to VPA.

## Materials and methods

### Fungal strains, media, and chemicals

The fungal clinical and laboratory strains used in this study are listed in the Tables S1, S2, respectively. *C. albicans* and other yeast strains were routinely maintained at 30°C on YPD (1% yeast extract, 2% peptone, 2% dextrose, with 50 mg/ml uridine) or synthetic complete (SC; 0.67% yeast nitrogen base with ammonium sulfate, 2.0% glucose, and 0.079% complete supplement mixture) or RPMI (RPMI-1640 with 0.3 g/L-glutamine) media. Acidic pHs used for VPA susceptibility were obtained using hydrochloric acid.

Valproic acid (VPA; Sigma-P4543) was dissolved in sterile water (50 mg/ml). Standard antifungals used for VPA-synergy assessment are: Fluconazole (FCZ; Sigma-F8929), Caspofungin (CSP; Sigma-SML0425), Voriconazole (VCZ; Sigma-PZ0005), Amphothericin B (AMB; Sigma-A488), Itraconazole (ITZ; Sigma-I6657), Clotrimazole (CTZ; Sigma-C6019), Teroconazole (TCZ; Toronto Research Chemicals-T110600), Miconazole (MCZ; Sigma-PHR1618), Terbinafine (TRB; Sigma-T8826), Nystatin (NST; Sigma-N4014), and Micafungin (MCF; McKesson Canada-205666). Antifungals were prepared using DMSO for Amphothericin B (30 mg/ml), Fluconazole (300 mg/ml), Terbinafine (10 mg/ml), Clotrimazole (9 mg/ml), Nystatine (5 mg/ml), Miconazole (30 mg/ml), Terconazole (1 mg/ml); water for caspofungin (10 mg/ml), Voriconazole (10 mg/ml), Micafungin (10 mg/ml), and chloroform for Itraconazole (50 mg/ml).

### VPA susceptibility and time-kill assays

The pH-dependant effect of VPA on *C. albicans* was evaluated as follows: The reference clinical strain SC5314 was grown overnight in YPD medium at 30°C in a shaking incubator. Cells were then resuspended in fresh SC at an optical density at 595 nm (OD_595nm_) of 0.05. The pHs of SC media were adjusted using sodium hydroxide or hydrochloric acid for alkaline and acidic pHs, respectively. A total volume of 99 μl *C. albicans* cells was added to each well of a flat-bottom 96-well plate in addition to 1 μl of the corresponding stock solution of VPA. Plates were incubated in a Sunrise-Tecan plate reader at 30°C with agitation and OD_595nm_ readings were taken every 10 min over 24 h. Experiments were performed in triplicate, and average values were used for analysis. VPA effect on other fungal species at acidic pH was performed in a similar fashion.

The Minimal Inhibitor Concentration (MIC) was determined following the CLSI recommendations (CLSI, 2008). Briefly, 50 μl of VPA or standard antifungals at two-fold the final concentration prepared in RPMI was serially diluted in flat-bottom 96-well plates (Costar-Corning) and combined with 50 μl of an-overnight culture of *C. albicans* and other yeasts at 10^4^ cell/ml. Plates were incubated at 30°C with shaking and OD_595nm_ readings were taken after 24 h using the Sunrise-Tecan plate reader. The MIC was determined as the first well with growth reduction of >10% based on OD_595nm_ values in the presence of VPA or conventional antifungals as compared to untreated control cells. Time-kill was performed as described by Sanglard et al. (2003). Briefly, *C. albicans* SC5314 strain cultures were grown in RPMI pH 4.5 at 30°C under shaking in the presence of different concentration of VPA for defined time periods (6, 24, and 48 h). Fractions of cultures were removed at each exposition time and the colony forming units (CFU) counts were ensured by serial dilution in YPD-agar.

### Synergism assay

Evaluation of synergistic interactions between VPA and standard antifungals was performed using RPMI-1640 medium buffered at pH 4.5. Synergism was assessed by calculating the fractional inhibitory concentration (FIC) index as described by Epp et al. (2010). The FIC index was calculated as follows: (MIC of VPA in combination/MIC of VPA alone) plus (MIC of a standard antifungal in combination/MIC of a standard antifungal alone).

### Biofilm formation and XTT reduction assay

Biofilm formation and XTT (2,3-bis(2-methoxy-4-nitro-5-sulfo- phenyl)-2H-tetrazolium-5-carboxanilide) assays were carried out as previously described by Askew et al. (2011). Overnight YPD cultures were washed three times with PBS and resuspended in fresh RPMI supplemented with L-glutamine (0.3 g/l) to an OD_595nm_ of 1. *C. albicans* yeast cells were allowed to adhere to the surface of 96-well polystyrene plate for 3 h at 37°C in a rocking incubator. Non-attached cells were washed from each well three times with PBS and fresh RPMI supplemented with VPA was added for 24 h at 37°C for biofilm formation. The plates were then washed and fresh RPMI supplemented with 100 μl of XTT-menadione (0.5 mg/ml XTT in PBS and 1 mM menadione in acetone) was added. After 3 h incubation on the dark at 37°C, 80 μl of the resulting colored supernatants were used for colorimetric reading (OD_490nm_) to assess metabolic activity of biofilms. A minimum of four replicates were at least performed.

### Vaginal epithelial cell damage assay

Damage of vaginal epithelial cells was assessed using the lactate dehydrogenase (LDH) cytoxicity detection kit (Sigma) based on the release of LDH in the surrounding medium following the manufacturer's protocol. VK2/E6E7 (ATCC- CRL-2616) vaginal epithelial cell line was grown on a keratinocyte-serum free medium (supplemented with 0.1 ng/ml recombinant epidermal growth factor and 50 μg/ml bovine pituitary extract) as a monolayer to 95% confluency on a 96-well culture plate and incubated at 37°C with 5% CO_2._ VK2/E6E7 cells were infected with 2 × 10^4^ of *C. albicans* SC5314 blastospores for 24 h. A total of 100 μl supernatant was removed from each experiment and LDH activity in this supernatant was determined by measuring the absorbance at 490 nm (OD_490nm_). LDH activity were calculated as the mean of, at least, three independent biological replicates.

### Genetic screen for VPA-sensitive mutants

A total of 2371 mutants from the transcription factors (Homann et al., 2009) (365 strains), transcriptional regulators (Vandeputte et al., 2012) (509 strains), kinases (Blankenship et al., 2010) (165 strains), and generalist collections (Noble et al., 2010) (1,332 strains) were screened for VPA-sensitivity. These mutant libraries were obtained from the Fungal Genetics Stock Center (FGSC). With the exception of the kinase collection where genes were disrupted by transposon insertions, mutants of the other collections were created through gene deletion of the complete ORF. In most cases and for each gene, at least two independent transformants were screened. Mutant strains were grown overnight in SC with pH 4.5 on flat-bottom 96-well and were plated on SC-agar pH 4.5 medium with or without VPA (50 μg/ml) using a 96-well blot-replicator. Mutants exhibiting more than fold-fold growth reduction based on colony diameter were compiled together in a 96-well plate and their sensitivity were confirmed to different concentration of VPA (10, 50, and 100 μg/ml) following the same procedure. Mutant strain with established VPA sensitivity were individually reconfirmed by serial dilution spot assay. A complete listing of VPA-sensitive mutants is shown in Table S3. The overrepresentation of specific GO terms associated with the function of gene required for VPA tolerance was determined with GO Term Finder using a hypergeometric distribution with multiple hypothesis correction (http://www.candidagenome.org/cgi-bin/GO/goTermFinder) (Inglis et al., 2012). Descriptions related to gene function in Table S3 were extracted from CGD (Candida Genome Database) database (Inglis et al., 2012).

### Confocal microscopy and vacuole integrity

*C. albicans* vacuole integrity was assessed using the lipophilic vacuole membrane dye MDY-64 (Molecular probes, Fisher Scientific) following the manufacturer's recommended procedure. Briefly, cells were grown overnight on RPMI liquid medium with pH 4.5 at 30°C. Cells were pelleted and washed twice with fresh RPMI pH 4.5 and resuspended in the same medium at an OD_595_ of 0.1. VPA was added at different concentrations (10, 50, and 100 μg/ml). Cells were incubated for 2 h at 30°C under agitation. Aliquots were taken from VPA-treated and non-treated cultures and the MDY-64 was added at a final concentration of 10 μM. Cells were incubated at room temperature for 3 min prior to confocal microscopy visualization. Images were acquired with a 1.3-numerical-aperture (NA) 63x objective on a Leica DMI6000B inverted microscope connected to a Hamamatsu C9100-13 camera.

Pan1-green fluorescent protein (GFP), End3-GFP and LIFEACT-GFP (Epp et al., 2013) were visualized using confocal microscopy as follow: an overnight culture was diluted in SC supplemented with 10 or 50 μg/ml VPA to an OD_595nm_ of 0.05 and grown for four generations at 30°C under agitation. Cells were imaged as described for the vacuole staining experiments.

## Results

### Antifungal activity of VPA is pH-dependant

**Figure 1:**
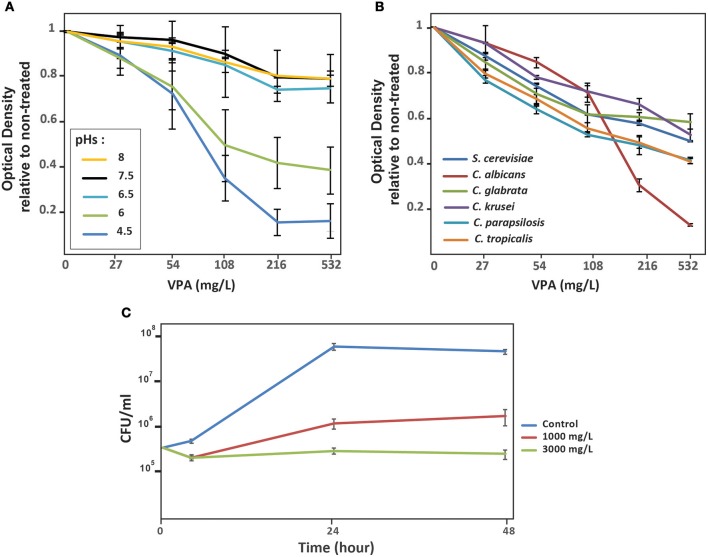
*In vitro* antifungal activity of valproic acid is pH-dependant. **(A)** Effect of different pHs on antifungal activity of VPA. The *C. albicans* SC5314 strain was grown in SC medium with different pH (4.5–8) supplemented with different concentration of VPA. SC5314 strain was grown at 30°C and OD_595nm_ reading was taken after 24 h of incubation. ODs measurement for each VPA concentration is the mean of triplicate. **(B)** VPA inhibit the growth of non-*albicans* Candida species. *C. glabrata, C. parapsilosis, C. tropicalis, C. krusei* in addition to *S. cerevisiae* were grown in SC medium pH 4.5 with different concentration of VPA. OD_595nm_ reading was taken after 24 h of incubation at 30°C under agitation. **(C)** Time-kill curve demonstrating the fungistatic activity of VPA. *C. albicans* SC5314 strain was exposed to two different concentrations (1,000 and 3,000 μg/ml) at different times (6, 24, and 48). CFUs were calculated as described in the method section.

Antifungal activity of VPA on *C. albicans* was evaluated by monitoring OD_595nm_ of cultures exposed for 24 h to increased concentration of VPA in SC media at different pHs. VPA exerted an inhibitory effect that was exaggerated in acidic pH (Figure 1A). Antifungal activity of VPA was also assessed in other clinically relevant Candida species including *C. glabrata, C. tropicalis, C. parapsilosis*, and *C. krusei* in addition to the yeast *S. cerevisiae*. The obtained data demonstrates that VPA inhibited the growth of all tested fungal species, with *C. albicans* exhibiting higher sensitivity at elevated VPA concentrations (>216 μg/ml) (Figure 1B). Thus, VPA is a potent antifungal compound against *C. albicans* at acidic pH.

To test whether VPA had fungistatic or fungicidal activity on *C. albicans* at acidic pH, time-kill curve assays were performed. Two high concentrations of VPA which correspond to 125x (1,000 μg/ml) and 375x (3,000 μg/ml) of the MIC for the *C. albicans* reference strain SC5314 (Table 1) were tested. VPA exhibited a concentration-independent fungistatic activity (Figure 1C). Lower VPA concentrations ranging from 7.8 (MIC for the SC5314 strain) and 500 μg/ml were also tested and the obtained results demonstrates a similar fungistatic activity (result not shown).

**Table 1:** *In vitro* activity of valproic acid (MIC) on *C. albicans* antifungal sensitive and resistant strains.

**Isolates**	**Description**	**MIC (**μ**g/ml)**	
		**pH 7**	**pH 4.5**
***Candida albicans***			
SC5314	Azole-sensitive	>250	7.8
5457	Azole-sensitive	>250	15.6
5833	Azole-sensitive	>250	15.6
5674	Azole-resistant	>250	7.8
6692	Azole-resistant	>250	15.6
S2	Azole-resistant	>250	7.8
S1	Azole-sensitive	>250	3.5
F5	Azole-resistant	>250	3.5
G5	Azole-resistant	>250	7.8
DPL-1007	Echinocandin-resistant	>250	3.5
DPL-1008	Echinocandin-resistant	>250	7.8
DPL-1009	Echinocandin-resistant	>250	15.6
DPL-1010	Echinocandin-resistant	>250	7.8
HDQ-RP1	Echinocandin-resistant	>250	7.8
HDQ-RP2	Azole-resistant	>250	15.6

### Antifungal activity of VPA against azole- and echinocandin-resistant strains

Since VPA was highly potent against *C. albicans*, we wanted to test whether its antifungal activity can be expanded to other clinically sensitive and resistant strains of this yeast. Several azole-resistant strains with different resistant mechanisms, were selected (Table S1) in addition to echinocandin-resistant isolates. A total of four sensitive and 11 resistant strains (six azole- and five echinocandin-resistant strains) were examined using broth microdilution assay as specified by CLSI at both neutral or acidic pHs. The sensitivity of *C. albicans* isolates to VPA was pH-dependant and MICs ranged from 3.5 to 15.6 μg/mlfor both resistant and susceptible strains (Table 1). The range of MICs was also similar when comparing azole-resistant and echinocandin-resistant clinical strains separately (3.5–15.6 μg/ml). Overall, these results demonstrate that VPA may be of use to tackle therapeutic limitations related to acquired clinical resistance of *C. albicans*. Furthermore, comparable VPA-sensitivity in susceptible and resistant strains indicates that the mechanisms that confer resistance to azoles and echinocandins are distinct from those that may cause VPA resistance.

### Valproic acid attenuate damage of vaginal epithelial cells caused by *C. albicans*

**Figure 2:**
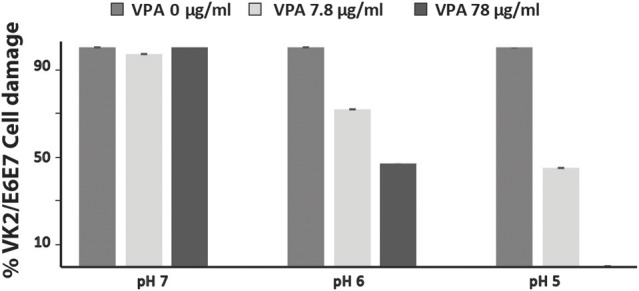
Valproic acid attenuate damage of vaginal epithelial cells caused by *C. albicans*. Damage of human epithelial vaginal cell line VK2/E6E7 infected by *C. albicans* SC5314 strain was assessed using LDH release assay. For each pH, cell damage was calculated as percentage of LDH activity of VPA-treated experiment relatively to that of the control experiment (*C. albicans* invading VK2/E6E7 cells in the absence of VPA). Results are the mean of three independent replicates.

To verify whether VPA exerts protective antifungal activity during host cell invasion, interaction of *C. albicans* with the human epithelial vaginal cell line VK2/E6E7 were performed as described in the method section. *C. albicans*-mediated damage of VK2/E6E7 cells were quantified based on the LDH release. Two different concentrations of VPA (7.8 and 78 μg/ml) corresponding to the MIC and 10x MIC for *C. albicans* SC5314 strain were used. In accordance with our *in vitro* data, the VPA had no significant protective effect at pH 7 (Figure 2). At pH 5, 7.8, and 78 μg/ml of VPA prevented 55 and 100% of VK2/E6E7 damage, respectively, as compared to the control. Intermediate protective activity was perceived at pH 6 where 28 and 52% damage reduction was obtained with 7.8 and 78 μg/ml of VPA, respectively. In support of *in vitro* data, these results demonstrate that VPA confers a protective antifungal activity during the invasion of vaginal epithelial cells.

### VPA acts synergistically with terbinafine in both susceptible and resistant strains

Different standard antifungals used against *C. albicans* and other human fungal pathogens were screened to identify drugs that could potentiate the anti-Candida activity of VPA. Interactions of VPA with other 11 antifungal agents including azoles (Fluconazole, Voriconazole, Itraconazole, clotrimazole, Terconazole, and Miconazole), polyenes (Amphothericin B and Nystatin), echinocandins (Caspofungin and Micafungin), and the allylamine, Terbinafine were tested. Based on the appreciation of the FIC index in the clinical strain SC5314, VPA was found to exhibit an apparent synergistic interaction with terbinafine (Table 2; FIC index < 0.5). VPA-terbinafine combinations were also synergistic in azole and echinocandin resistant clinical strains (Table 2).

**Table 2:** Synergistic interaction of valproic acid with the allylamine antifungal, terbinafine on sensitive, and azole- and echinocandin-resistant strains.

**Strain name**	**MIC (**μ**g/ml)**				**FIC index**
	**Alone**		**In combination**		
	***TRB***	***VPA***	***TRB***	***VPA***	
SC5314	4	7.8	0.125	1.9	0.271
5674	72	7.8	9	1.9	0.365
F5	144	4	18	1	0.375
DPL1008	4	7.8	0.063	1.9	0.255

### VPA inhibits biofilm formation in both susceptible and resistant strains

**Figure 3:**
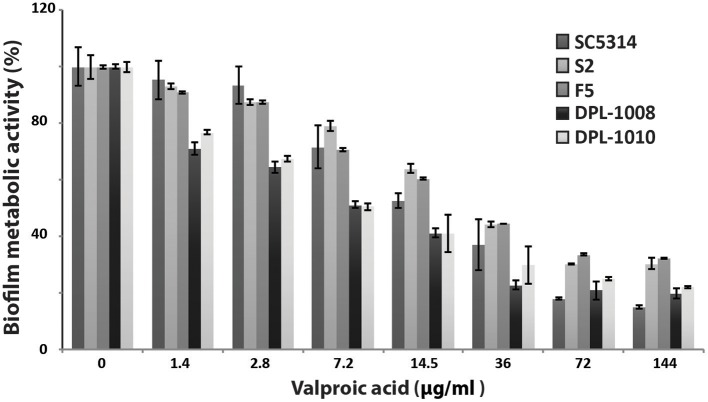
Anti-biofilm activity of valproic acid. The effect of VPA on biofilm formation was evaluated using the metabolic colorimetric assay based on the reduction of XTT at pH4. Sensitive (SC5314) and azole- (S2 and F5), and echinocandin-resistant (DPL-1008 and DPL-1010) *C. albicans* strains were tested. Results represent growth inhibition (%) and are the mean of at least three independent replicates.

The effect of VPA on biofilm formation was evaluated using the metabolic colorimetric assay based on the reduction of XTT at acidic and neutral pHs. At neutral pH, no VPA anti-biofilm activity was noticed of all tested concentrations for the *C. albicans* SC5314 reference strain (not shown). In contrast, at pH 4.5, biofilm inhibition was apparent at 1.44 μg/ml of VPA with ~5% of inhibition as compared to the control (Figure 3). The MIC of VPA on the SC5314 strain was evaluated at 7.2 μg/ml. The effect of VPA on biofilm formation was also tested in two azole-resistant strains (S2 and F5) with different resistance mechanisms in addition to two echinocandin-resistant isolates (DPL-1008 and DPL-1010). As for the SC5314 sensitive strain, the four resistant strains exhibited a clear reduction in metabolic activity at 1.44 μg/ml of VPA (Figure 3). The MIC values for the azole-resistant strains were similar (2.88 μg/ml VPA) and slightly decreased as compared to the SC5314 susceptible strain. The two echinocandin-resistant strains DPL-1008 and DPL-1010 were highly sensitive to VPA as compared to other strains and their MIC was noticed at 1.44 μg/ml of VPA. These results demonstrate that, in addition to its antifungal activity on planktonic cells, VPA is also active on sessile forms of *C. albicans* at acidic pH.

### Mutants defective in vacuolar functions are hypersensitive to VPA

To gain insight into the mechanism of action of VPA associated with its antifungal property, a comprehensive regulatory and generalist mutant collections of *C. albicans* were screened for their sensitivity to VPA. Among the **947** unique mutants that were screened, 55 were confirmed to be hypersensitive to VPA (Table S3). To identify the functional categories that are associated with mutations affecting VPA susceptibility, we performed gene ontology (GO) enrichment analysis. Our data demonstrated that VPA sensitive mutants are defective in genes related primarily to vacuole transport (*p* = 1.72e-08) and organization (*p* = 8.86e-09) (Table 3, Table S4). This include mutants of vacuolar protein sorting (*vps15, vps34, vps64*, and *ypt72*), proteins associated with the retromer complex (*pep7* and *pep8*), and proteins required for vacuole inheritance, and organization (*cla4, pep12, vam6, vps41*, and *pep12*). Requirement of vacuolar functions for VPA tolerance was also reported previously in *S. pombe* (Zhang et al., 2013) and *S. cerevisiae* (Deranieh et al., 2015) where genome-wide screens demonstrated that retromer complex and vacuolar ATPases, respectively, were associated with VPA sensitivity. Taken together, our chemogenetic screen provides a rational for mechanistic investigation into the effect of VPA on fungal vacuole.

**Table 3:** Gene function and biological process associated with VPA-sensitivity.

**Functional group of genes**	**Gene name**	***p*-value^a^**
Vacuole organization	*APM1, CLA4, NEO1, PEP7, PEP12, PKH2, PRB1, VAM6, VPS15, VPS34, VPS41, YPT72*	8.86E-09
Vacuolar transport	*APM1, ARL1, PEP7, PEP8, PEP12, PKH2, RCY1, RHB1, VAM6, VPS15, VPS34, VPS41, VPS64, YPQ1, YPT72*	1.72E-08
Vesicle-mediated transport	*APM1, ARL1, CLA4, GYP1, KIN2, OSH3, NEO1, PEP7, PEP8, PEP12, RCY1, RHB1, VAM6, VPS15, VPS34, VPS41, YPT72*	2.21E-08
Vacuole inheritance	*CLA4, PEP7, PEP12, VPS15, VPS41, YPT72*	1.84E-06
Cellular response to extracellular stimulus	*APM1, CLA4, KAR3, KIC1, KRE5, OCH1, PEP7, PEP8, PRB1, RAV2, RHB1, SSU1, UBR1, VPS34, VPS41*	1.03E-06

*Gene ontology analysis was performed using GO Term Finder*.

a *The p-value was calculated using hypergeometric distribution, as described on the GO Term Finder website (Inglis et al., 2012)*.

### VPA alters vacuole morphology

**Figure 4:**
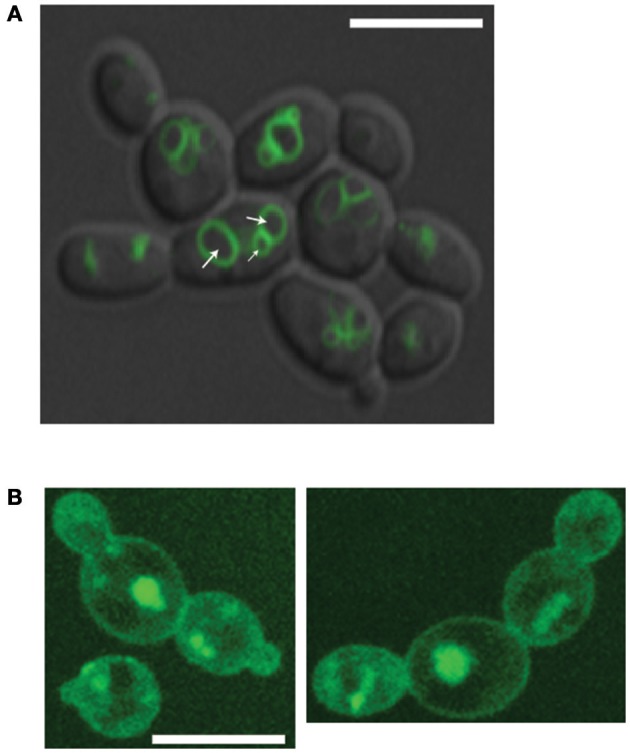
Valproic acid alters vacuolar morphology. *C. albicans* SC5314 strains was grown in RPMI pH 4.5 in the absence **(A)** or presence of 50 μg/ml of VPA **(B)** and stained for 3 min with the vacuole membrane marker, MDY-64. Cells were visualized using confocal microscopy. The white arrows indicate representatively intact vacuole lumens. Fluorescence PMT gain were increased five times for VPA-treated cells due to low incorporation of MDY-64. Bars, 8 μm.

Our chemogenetic screen demonstrated clearly that *C. albicans* sensitivity to VPA were exaggerated in mutant of vacuolar transport, organization and inheritance. The requirement of intact vacuolar pathways for VPA tolerance suggests that VPA might alters the function and/or the integrity of the vacuole. To verify this hypothesis, the integrity of *C. albicans* vacuoles were assessed using the vacuole membrane marker, MDY-64, in cells treated or not with different concentrations of VPA at pH 4.5. A dominant fraction of non-treated cells internalized the MDY-64 dye and exhibited well-structured vacuoles with two to four compartments comprising discernable lumens (Figure 4A). However, cells treated with either 10 or 50 μg/ml of VPA displayed an altered vacuole structure with a foamy fluorescence pattern and indistinguishable lumens (Figure 4B). These findings suggest that VPA affect the morphology and the integrity of vacuoles in *C. albicans*.

### VPA-induced vacuolar phenotypes are not a consequence of endocytosis, actin filaments perturbations, or inositol depletion

Perturbation of vacuoles by VPA might be either a direct consequence where VPA act *in situ* on vacuole or by impacting indirectly other process required for proper vacuole biogenesis and organization. Indeed, vacuolar integrity and homeostasis depends on the proper functioning of different cellular processes including actin filaments organization (Eitzen et al., 2002), endocytosis (Michaillat and Mayer, 2013), and the phosphatidylinositol phosphate signaling (Michell et al., 2006). Recent work by Deranieh et al. (2015) indicated that VPA led to cellular depletion of inositol, which disrupts the vacuolar phosphoinositide, PI(3,5)P2 (Phosphatidylinositol 3,5-bisphosphate) homeostasis and consequently compromise vacuole morphology. In this regard, we checked whether VPA-mediated vacuole alteration can be bypassed by inositol supplementation or deprivation. Our data demonstrate that the altered vacuole phenotype was not influenced by lack or excess of inositol (data not shown), suggesting that the probable VPA-induced inositol depletion in *C. albicans* is unlikely to account for the toxicity of this compound under our conditions.

To check whether the vacuole alteration caused by VPA is related to a defect in endocytosis or actin filament organization, we have used a Pan1- and End3-GFP fusions as clathrin-coated vesicles markers, and LIFEACT-GFP (Epp et al., 2013) to monitor actin patches and cables. VPA treatment did not cause any apparent alteration of the organization of actin filaments or endocytic vesicles (Figure S1). Taken together, these results support that the direct alteration of vacuole is the cellular mechanism underlying the antifungal activity of VPA.

## Discussion

*Candida* pathogenic species are adapted to survive in different acidic environments inside their host such as the vagina, inflammatory foci like abscesses (Park et al., 2012) and phagolysosomes of neutrophils and macrophages (Erwig and Gow, 2016). In such acidic condition, several studies demonstrated that the *in vitro* activity of standard antifungals is compromised as evidenced by the increase of their MICs (Marr et al., 1999; Pai and Jones, 2004; Danby et al., 2012). In the current study, we demonstrated that the antifungal activity of the VPA, a histone deacetylase inhibitor and the widely prescribed as antipsychotic, is potentiated at acidic pH that resemble to that of host niches cited above. We also demonstrated that VPA potentiates the antifungal activity of the widely prescribed terbinafine at acidic pH. In this regard, VPA, alone or with terbinafine, may be useful against fungal vaginosis caused primarily by *C. albicans*. VPA was also found to be effective against both echinocandin- and azole-resistant strains suggesting that this molecule represents an alternative solution to circumvent VVC or recurrent VVC caused by *C. albicans* strains that are resistant to standard antifungals. In the current study, VPA were also potent against *C. albicans* biofilm in a similar fashion as for planktonic cells and for both sensitive and clinical resistant strains. As for vaginal bacterial pathogens, *C. albicans* is able to form infective biotic biofilms on the vaginal mucosal surfaces (Harriott et al., 2010). Due to the fact that biofilm growth is impervious to all conventional antifungals, and since efficiency of these drugs is compromised at acidic pH, VPA may represent thus a promising alternative for antibiofilm therapy.

Importantly, this work supports a direct clinical repurposing of VPA as an antifungal against VVC or recurrent VVC due to the fact that its safety profile has been extensively characterized *in vivo* over the past decades of its clinical use in systemic forms as anticonvulsant (Lagace et al., 2004) or anticancer (Gupta et al., 2013). VPA had also a broad therapeutic safety margin when used topically (Choi et al., 2013). It does not cause skin irritations such as erythema and edema and had no toxicity to different human cells including keratinocytes, fibroblasts, and mast cells (Choi et al., 2013). In the current work, we also find that VPA did not impair the growth and the integrity of the vaginal epithelial cells VK2/E6E7 as judged by the LDH cytotoxicity assay (Figure S2). While a whole animal vaginal model is required to confirm that VPA does not cause vaginal irritations, the aforementioned studies are supportive of a safe use of VPA topically against VVC.

It is intriguing that the antifungal activity of VPA was acidic pH-dependant. This could be explained by the chemical nature of VPA, which is an eight-carbon branched-chain acid with proprieties of weak acid (pKa 4.8). Low pH is expected to decrease its ionization state and increase its liposolubility, which in turn may facilitate the passage through the plasma membrane and its accumulation in the cells. Future structure-guided medicinal chemistry approach by introducing structural changes in VPA that can lead to beneficial biological activity in a pH-independent manner will allow expanding the potential use of this molecules form VVC and recurrent VVC to treat oral *C. albicans* infections and even systemic candidiasis.

In the current study, we undertook a chemogenetic screen to delineated biological process that underlies VPA-sensitivity in *C. albicans*. This screen enables the identification of different vacuole-related functions as being required to tolerate VPA and provide thus a rational to examine the effect of this molecule on fungal vacuole. Our data demonstrates clearly that VPA antifungal activity is a consequence of the impairment of vacuole integrity and illuminate thus a previously unappreciated mechanism of action of this drug. Recent work in *S. cerevisiae* indicates that cellular depletion of inositol by VPA disrupts the vacuolar phosphoinositide, PI3,5P2 homeostasis which compromise the function of V-ATPase activity and proton pumping (Deranieh et al., 2015). This V-ATPase phenotype was rescued by supplementing the growth medium by inositol. Despite the requirement of V-ATPases to tolerate VPA in *S. cerevisiae*, the authors did not report any alteration of the vacuolar morphology by VPA as seen in our investigation. Furthermore, the vacuole defects in *C. albicans* were not recovered by adding inositol to the growth medium suggesting that VPA may act via a different mechanism in this pathogenic yeast. Similarly, in *S. pombe*, genetic screens revealed that mutant of genes operating in Golgi-endosome membrane trafficking and vacuole retromer complex were hypersensitive to VPA (Miyatake et al., 2007; Ma et al., 2010; Zhang et al., 2013), however, no apparent alteration of vacuole was seen in this yeast model.

Regardless of the exact vacuolar process that is targeted by VPA, our study reinforces the fact that pharmacological perturbation of vacuole leads to fungal growth inhibition and is protective for host cells. Different *C. albicans* vacuolar proteins has been previously characterized and linked to the ability to infect the host and to control different virulence traits including biofilm formation, filamentation, and resistance to antifungals. This include for instance vacuolar membrane and cytosolic V-ATPases (Vma2, Vma3, and Vph1) (Patenaude et al., 2013; Rane et al., 2013, 2014), proteins mediating vesicular trafficking to the vacuole (Pep12, Vps11, and Vps21) (Palmer et al., 2005; Johnston et al., 2009; Palanisamy et al., 2010; Wachtler et al., 2011) and the vacuolar calcium channel, Yvc1 (Wang et al., 2011). This makes the vacuole an ideal therapeutic target to manage fungal infections. However, the functional resemblance of fungal vacuoles with their human counterpart organelle, lysosomes, raises uncertainty regarding their druggability. Indeed, while the two V-ATPase inhibitors bafilomycin A1 and concanamycin A from *Streptomyces*, exhibit a potent activity against *C. albicans* they also compromise the activity of the mammalian V-ATPases (Olsen, 2014). Meanwhile, the fungal vacuoles had distinctive proteins such as the V0-ATPase subunit with no apparent human homologs that could be specifically targeted for pharmacological interventions in the treatment of fungal infections. In this regard, we demonstrate that VPA had no cytotoxicity on vaginal epithelial cells at concentrations above 10 times the MIC of *C. albicans* suggesting that VPA-mediated vacuole alteration is fungus-specific (Figure S2).

In conclusion, we have shown that VPA is a potent antifungal at acidic pH and consequently an attractive therapeutic molecule against vulvovaginal candidiasis. We have also described an unreported effect of VPA on the structural integrity of fungal vacuoles which might be the main cause of its cytotoxicity.

## Author contributions

AS designed the experiments; JC, FT, CG performed the experiments; JC, FT, CG, HW, RP, and AS analyzed the data; AS and JC wrote the manuscript with the help of all authors.

### Conflict of interest statement

A provisional patent application for an antifungal vaginal gel formulation related to this work (Patent number US 62/532,603) has been filled by Université Laval. The authors declare that the research was conducted in the absence of any commercial or financial relationships that could be construed as a potential conflict of interest.
