# Supplementary material for: pH-Dependant Antifungal Activity of Valproic Acid against the Human Fungal Pathogen Candida albicans
Source: Front Microbiol. 2017 Oct 9;8:1956. doi: 10.3389/fmicb.2017.01956 (PMC5640775; doi:10.3389/fmicb.2017.01956)
Supplement: Supplementary file 1 [file Table1.pdf]

Table S1. Fungal strains used in this study.

Name	Description	Reference
Candida albicans		
SC5314 (ATCC-MYA-2876)	*C. albicans* wild-type reference strain.	(Fonzi and Irwin, 1993)
5457	Azole-susceptible clinical strain isolated from throat	(Saidane et al., 2006)
5833	Azole-susceptible clinical strain isolated from mouth	(Saidane et al., 2006)
5674	Azole-resistant clinical strain (overexpressing the ABC-transporters Cdr1 and Cdr2, and the phosphatidylinositol transfer protein, Pdr16) isolated from mouth	(Saidane et al., 2006)
6692	Azole-resistant clinical strain (overexpressing the MFS-transporter MDR1 and had a gain-of-function mutation on the transcription factor, Mrr1) isolated from mouth	(Saidane et al., 2006;Dunkel et al., 2008a)
S1	Fluconazole-susceptible clinical strain isolated from patient with AIDS	(Franz et al., 1999;Dunkel et al., 2008b)
S2	Fluconazole-resistant clinical strain isolated from patient with AIDS (resistance related to gain-of-function mutation of the transcription factor, Upc2 and Erg11 overexpression)	(Franz et al., 1999;Dunkel et al., 2008b)
F5	Azole-resistant clinical strain (overexpressing MDR1 and harbouring a gain-of-function mutation on the transcription factor, Mrr1) isolated from oral cavity from patient with AIDS	(Franz et al., 1998;Morschhauser et al., 2007)
G5	Azole-resistant clinical strain (overexpressing MDR1 and harbouring a gain-of-function mutation on the transcription factor, Mrr1) isolated from oral cavity from patient with AIDS	(Franz et al., 1998;Morschhauser et al., 2007)
DPL-1007	Clinical isolate resistant to echinocandin harbouring the F641S mutation on the beta-(1,3)-glucan synthase, Fks1p	(Jimenez-Ortigosa et al., 2014)
DPL-1008	Clinical isolate resistant to echinocandin harbouring the F645P mutation on the beta-(1,3)-glucan synthase, Fks1p	(Jimenez-Ortigosa et al., 2014)
DPL-1009	Clinical isolate resistant to echinocandin harbouring the F645Y mutation on the beta-(1,3)-glucan synthase, Fks1p	(Jimenez-Ortigosa et al., 2014)
DPL-1010	Clinical isolate resistant to echinocandin harbouring the F645F mutation on the beta-(1,3)-glucan synthase, Fks1p	(Jimenez-Ortigosa et al., 2014)

HDQ-RP1	Clinical isolate resistant to caspofungin from L'Hôtel-Dieu de Québec Hospital	-
HDQ-RP2	Clinical isolate resistant to fluconazole isolated from urine at L'Hôtel-Dieu de Québec Hospital	-
Saccharomyces cerevisiae		
BY4741 (ATCC 4040002)	*S. cerevisiae* laboratory reference strain used as genetic background for the systematic gene disruption project	(Brachmann et al., 1998)
Candida tropicalis		
MY070362	Clinical susceptible strain from INSPQ (Institut National de Santé Publique, Québec, Canada)	-
***Candida krusei* (*Issatchenkia orientalis*)**		
ATCC6258	ATCC reference strain. Isolated from the sputum of patient with bronchomycosis	(Rudek, 1978)
Candida parapsilosis		
ATCC90018	Reference susceptible strain used for CLSI antifungal susceptibility testing. Isolated from blood	(Espinel-Ingroff et al., 1992)

References

Brachmann, C.B., Davies, A., Cost, G.J., Caputo, E., Li, J., Hieter, P., and Boeke, J.D. (1998). Designer deletion strains derived from Saccharomyces cerevisiae S288C: a useful set of strains and plasmids for PCR-mediated gene disruption and other applications. *Yeast* 14**,** 115-132.

Dunkel, N., Blass, J., Rogers, P.D., and Morschhauser, J. (2008a). Mutations in the multi-drug resistance regulator MRR1, followed by loss of heterozygosity, are the main cause of MDR1 overexpression in fluconazole-resistant Candida albicans strains. *Mol Microbiol* 69**,** 827-840.

Dunkel, N., Liu, T.T., Barker, K.S., Homayouni, R., Morschhauser, J., and Rogers, P.D. (2008b). A gain-of-function mutation in the transcription factor Upc2p causes upregulation of ergosterol biosynthesis genes and increased fluconazole resistance in a clinical Candida albicans isolate. *Eukaryot Cell* 7**,** 1180-1190.

Espinel-Ingroff, A., Kish, C.W., Jr., Kerkering, T.M., Fromtling, R.A., Bartizal, K., Galgiani, J.N., Villareal, K., Pfaller, M.A., Gerarden, T., Rinaldi, M.G., and Et Al. (1992). Collaborative comparison of broth macrodilution and microdilution antifungal susceptibility tests. *J Clin Microbiol* 30**,** 3138-3145.

Fonzi, W.A., and Irwin, M.Y. (1993). Isogenic strain construction and gene mapping in Candida albicans. *Genetics* 134**,** 717-728.

Franz, R., Kelly, S.L., Lamb, D.C., Kelly, D.E., Ruhnke, M., and Morschhauser, J. (1998). Multiple molecular mechanisms contribute to a stepwise development of fluconazole resistance in clinical Candida albicans strains. *Antimicrob Agents Chemother* 42**,** 3065-3072.

Franz, R., Ruhnke, M., and Morschhauser, J. (1999). Molecular aspects of fluconazole resistance development in Candida albicans. *Mycoses* 42**,** 453-458.

Jimenez-Ortigosa, C., Paderu, P., Motyl, M.R., and Perlin, D.S. (2014). Enfumafungin derivative MK-3118 shows increased in vitro potency against clinical echinocandin-resistant Candida Species and Aspergillus species isolates. *Antimicrob Agents Chemother* 58**,** 1248-1251.

Morschhauser, J., Barker, K.S., Liu, T.T., Bla, B.W.J., Homayouni, R., and Rogers, P.D. (2007). The transcription factor Mrr1p controls expression of the MDR1 efflux pump and mediates multidrug resistance in Candida albicans. *PLoS Pathog* 3**,** e164.

Rudek, W. (1978). Esterase activity in Candida species. *J Clin Microbiol* 8**,** 756-759.

Saidane, S., Weber, S., De Deken, X., St-Germain, G., and Raymond, M. (2006). PDR16-mediated azole resistance in Candida albicans. *Mol Microbiol* 60**,** 1546-1562.
