# Supplementary figures and images for: pH-Dependant Antifungal Activity of Valproic Acid against the Human Fungal Pathogen Candida albicans
Source: Front Microbiol. 2017 Oct 9;8:1956. doi: 10.3389/fmicb.2017.01956 (PMC5640775; doi:10.3389/fmicb.2017.01956)

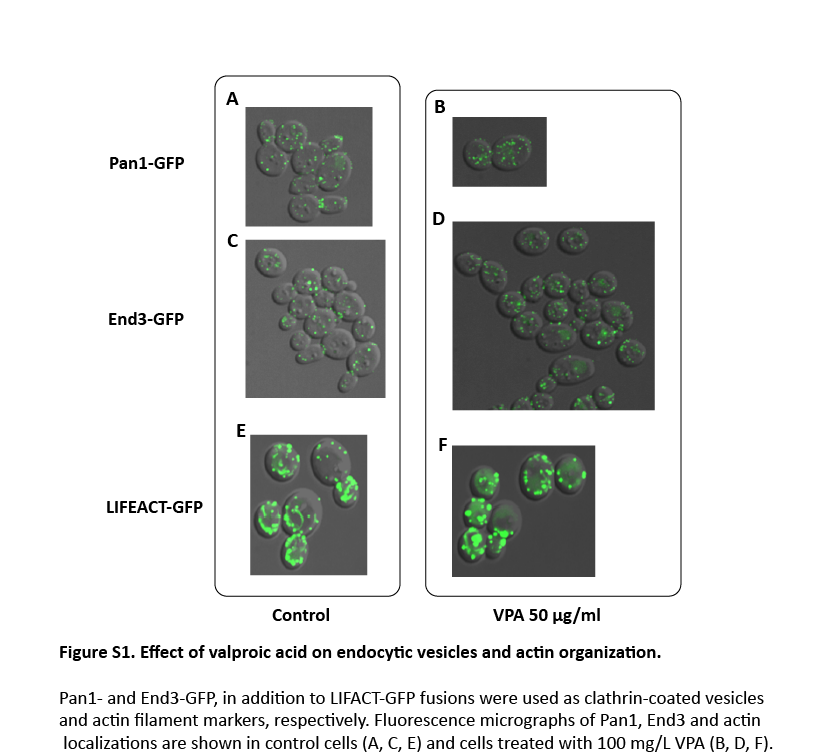

Supplement: Supplementary file 5 [file Image1.tif]

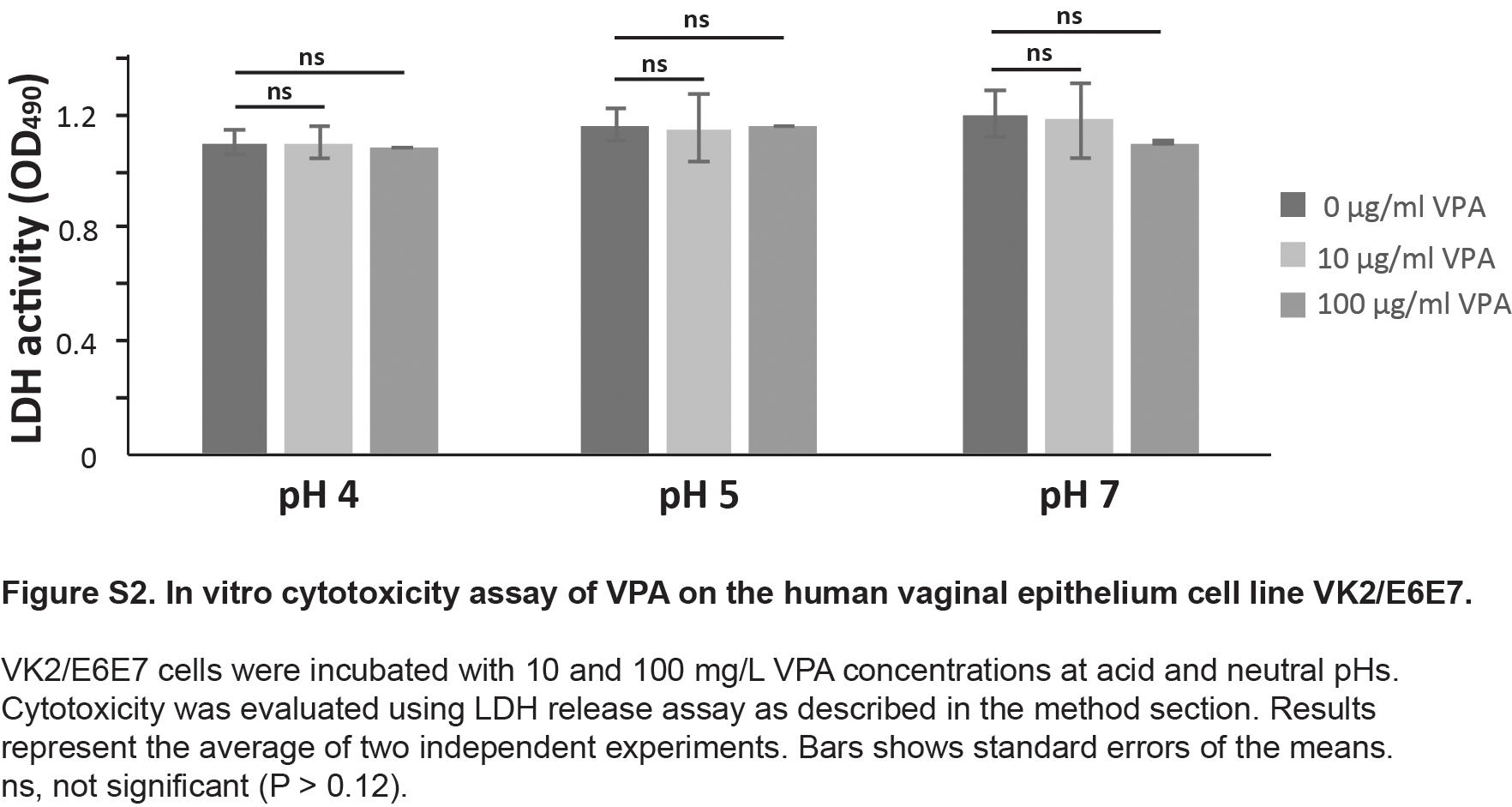

Supplement: Supplementary file 6 [file Image2.TIF]
